# Experiences of Adolescents with Obesity who Completed a Self-Weighing Feasibility Study

**DOI:** 10.21203/rs.3.rs-8321471/v1

**Published:** 2026-02-10

**Authors:** Charles B. Silverman, Aaron S. Kelly, Claudia K. Fox, Nancy E. Sherwood, Erica Urbina, Amy C. Gross, Carolyn T Bramante

**Affiliations:** University of Minnesota; University of Minnesota; University of Minnesota; University of Minnesota; University of Minnesota; University of Minnesota; University of Minnesota

## Abstract

**Background:**

Daily self-weighing is an evidenced-based weight-management strategy but has not been assessed in adolescents. We report results from qualitative exit interviews soliciting perspectives of participants and their parents following a single-arm feasibility study using smart scales connected to the electronic health record (EHR) among adolescents seeking obesity treatment.

**Methods:**

Adolescents seeking obesity treatment in a comprehensive pediatric weight management clinic were enrolled in a feasibility study assessing the use of smart scales connected to the clinic EHR. Inclusion criteria included age 12–18 years and body mass index ^3^95^th^ percentile for age and sex. Exclusion criteria included active eating disorders and severe depression/anxiety. At enrollment, participants received a one-time daily self-weigh suggestion and a handout about scale connection to the EHR. Interviews were transcribed, recorded, and conducted separately from parents unless requested otherwise.

**Results:**

The main theme was related to past weight-loss journey. Sub-themes included: intervention experience, parental involvement, being told by clinicians to weigh, and intervention impact. Most parents desired help connecting the app to the EHR. Most parents did not ask daily about weight status to not cause stress/anxiety. Some adolescents felt stressed when parents asked about weight status daily, some found it helpful. Most participants were never advised by their clinician to regularly self-weigh, but found daily self-weigh helpful. Most requested reminders to weigh from clinic and for feedback on weight between visits.

**Conclusions:**

Overall, adolescents with obesity reported self-weighing being helpful and most wanted some parent involvement. Most parents wanted additional technological support with scale set-up.

## Background

**Figure 1:**
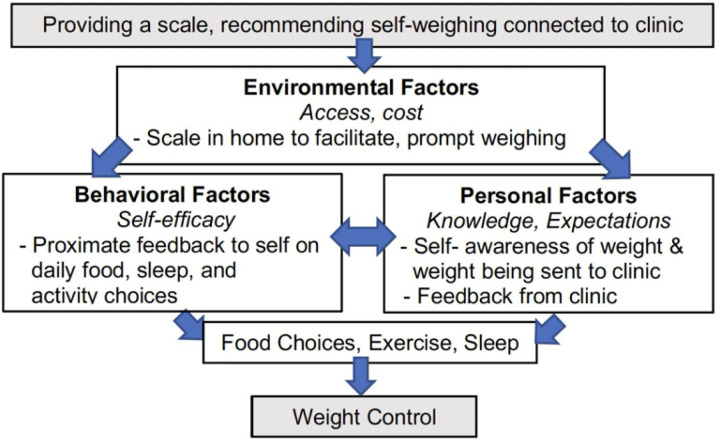
Conceptual model based on Social Cognitive Theory

Self-monitoring is a health-promotion behavior that has been studied in adults for managing a variety of chronic conditions. Self–monitoring is grounded in the Social Cognitive Theory, which states that self-monitoring improves self-efficacy and goal attainment.^1^ For obesity treatment, self-monitoring recommendations often include physical activity and dietary intake. Figure 1 adapts Albert Bandura’s social learning theory to self-monitoring of body weight for obesity treatment: environmental factors include physically having a scale to use; Behavioral factors include the number on the scale as information that is more proximate to current daily behaviors than a clinic weight in the future; personal factors include the knowledge that the clinic is also aware of the weights.

Having excess adiposity during adolescence can cause physical and psychological harm during adolescence as well as adulthood.^2–7^ Over 30% of adolescents in the US are projected to have obesity by the year 2050 obesity.^8^ Obesity is more prevalent in adolescents from low socioeconomic backgrounds, highlighting the importance of understanding obesity treatment options that are safe and effective, but also easily accessible.^9–11^ To our knowledge, daily self-weighing has not been prospectively studied in adolescents with obesity seeking obesity treatment. Our over-arching goal is to understand whether self-weighing would be a beneficial component to add to obesity treatment in adolescents, without causing stress or harm.

Daily self-monitoring of weight has been studied as self-monitoring tool for adults trying to lose weight.^12,13^ In adults, daily self-weighing has been found to improve weight loss and maintenance of weight loss compared to weekly weighing.^13,14^ The psychological impacts of weighing daily have been prospectively studied in adults showing that in most adults, this form of self-monitoring does not cause psychological harm.^15–17 18^ Previous studies in adolescents have shown mixed associations between self-weighing and psychological outcomes, but have not focused on adolescents with obesity seeking obesity treatment.^19^ A cross-sectional survey of adolescents who are overweight but not obese who had been in an obesity treatment program assessed self-weighing frequency.^20^ Adolescents who weighed at least once per week, compared to less frequent weighing, had an increased association with practicing healthy weight control practices, as well as an increased association with changing their behavior during the yearlong obesity treatment program.^20^ However, the impact of self-weighing in adolescents undergoing clinically-supervised obesity treatment has not been prospectively studied.^21,22^

The objective of this study is to present the results of qualitative interviews of adolescents who completed a self-weighing feasibility study. The self-weighing feasibility study was a prospective, single-arm study in which adolescents with obesity seeking obesity treatment were advised to weigh themselves daily. The results presented here are the qualitative experiences of these adolescents and their parents.

## Methods

### Study sample and design.

This study is a qualitative analysis of adolescent and parent perspectives on the use of self-weighing added to the clinical care of adolescents with obesity. In the parent study, adolescents participated in a single-arm feasibility study that assessed the ease of connecting blue-tooth scales to the electronic health record (EHR). All participants in the single-arm feasibility study were adolescents aged 12–17 with obesity who were seeking treatment at a tertiary care weight management clinic. A total of 29 adolescents completed the single-arm feasibility study. All the families who participated in the feasibility study received a one-time invitation to complete a 1:1 interview with a research staff member to better understand their experience with the scales and with self-weighing. Theme saturation was achieved after interviewing the 11 adolescents who responded to the invitation.

#### Human Ethics Declaration and Consent to Participate Declarations:

Every Parent/guardian provided informed consent to participate and adolescents provided assent to participate in the study. The study was approved by the University of Minnesota Institutional Review Board (IRB), STUDY00012679. The study was conducted according to the Declaration of Helsinki and according to Good Clinical Practice Guidelines. Clinical trial number not applicable.

##### The single-arm feasibility study

The single arm feasibility study involved using the patient-portal application to connect a blue-tooth enabled scale to the EHR using either Apple or Android phones. The participants and their families started this process in clinic with the research coordinator and completed the connection over the phone if needed. Participants were instructed that the primary aim of the pilot study was the feasibility of using an EHR-connected scale, but that weighing once per week for 12 weeks was a general recommendation for the pilot. The instructions during the self-weighing feasibility study were to weigh at a consistent time every time that they weighed (i.e. in the morning with a consistent amount of clothes on).

##### Qualitative Data Collection

The study team member conducting the interviews was not a member of their clinical team and was not the principal investigator. Adolescents and their parents were interviewed separately as some of the questions were about how much parental involvement the adolescents wanted for self-weighing. The participants were asked a set of questions designed to understand their thoughts about self-weighing. The interviewer was trained in qualitative methods for starting with open-ended questions. Table 1 includes a list of the question prompts.

The interviews were recorded and transcribed verbatim without any personal identifying information. Each participant was given a unique study ID number, and only the study ID was used as an identifier during the recorded interviews.

##### Coding

One research team member, with prior training on qualitative methods, was the primary coder. The initial coding procedure involved reading through all transcripts without working on organizing the transcripts on this first pass. The primary coder then organized the transcripts into paragraphs on related topics. Then the secondary coder further refined the paragraphs using a thematic analysis approach based on grounded theory.^23^ Under the third theme that arose, the coders looked for positive, neutral, and negative reactions.

## Results

### Participants

A convenience sample of 11 adolescent participants from the 29 participants in the single arm feasibility study participated in the qualitative interviews, and 9 parents participated (some parents had multiple children in the study). The mean age was 15.7 years, and the mean BMI was 39.7 kg/m^2^ at the time of the feasibility trial (BMI was not remeasured because the focus groups were conducted remotely). The self-identified gender of the adolescent participants was 9 female and 2 male; for the parents, 9 were female and 0 male.

From the coded transcripts, three main themes emerged: 1) the parents’ involvement with their child’s weight journey 2) the scale-connection experience; and 3) the adolescents’ experience with the weighing. Overall, the themes were similar for both the adolescents and the parents, and tracked closely to the interview guides for both parents and adolescents (Table 1). We present the themes with paraphrased text under each theme in the following paragraphs, and we present direct quotes in the Tables 2 and 3.

#### Emerging Themes

##### 1) The parents’ involvement with the adolescents’ weight journey

Most participants and parents had previously been told to not weigh frequently. Instead, they were told only to focus on how they looked and felt. During the single-arm feasibility study, some parents asked their children about their weight status, but most parents did not as they did not want to cause stress or anxiety. While some adolescents found it stressful when parents asked about their weight, others found it helpful or at least not stressful. Some adolescents felt that when their parents asked about their weight, they were asking because they were “fat”; however, they did not feel this way when the doctor asked about their weight. Some parents said that they did not ask their child about their weight, and some reported that their children voluntarily told them when they lost weight.

Before participation in the study began, adolescents recalled being excited and thinking studies like these are important because they might help prevent worsening childhood obesity. None of the adolescents had been told to weigh regularly by a primary care or weight management clinic, but rather were told by their general practitioner to not weigh themselves regularly because of concern that there are detrimental mental health side effects related to daily weight checks.

Before the study was conducted, parents reported that their children were excited about the technology aspect of the scale provided. Some parents were excited because they felt that self-weighing would help them track treatment progress. Some parents thought that daily weigh-ins were a good idea because they did not need to go to the doctor’s office to know their weight. Additionally, they felt self-weighing would be helpful because children could see fluctuations in their weight and think about what may be causing it. Some parents reported that this study could be improved if there was more accountability from clinic and if there was an educational aspect on how physical activity and eating habits are related into weight loss. Some parents also reported that they wanted access to their children’s weight records so they could encourage them to remember to weigh themselves.

##### 2) The intervention experience:

Technology was a large component of the intervention experience. About half of the adolescents (5 out of 11) had trouble setting up the app and synchronizing it with the EHR patient portal. Similarly, they had difficulty getting their weights to sync to the app and therefore gave up on regular weigh-ins. Some participants reported that more obvious reminders would have been helpful to remember to weigh in daily. Some of the adolescents (3 out of 11) reported that they had to open the app in order to get the notification to weigh in. While participants (6 out of 11) had trouble setting up the scale and wished it had been easier to set up, they thought it was generally very easy to use once set up. The main issue reported about the app after setup was successfully completed was that notifications were difficult to see.

Before participating, some parents were concerned that their child might become self-critical if their weight started going up or down. Some parents anticipated the study would be stressful for their children because they may forget to weigh. These parents suggested that their children weigh once a week and not every day. Some parents thought that daily weigh-ins were too much, did not see the benefit of weighing in every day, and felt like there needs to be other factors incorporated into sending weights to clinic (such as specific exercise or diet suggestions sent between clinic visits).

Over half of the adolescent participants thought it was helpful and encouraging to see their weight go down, and that regardless of weight change, knowing their weight at home improved their awareness of their bodies. Participants reported that the weighing made them more aware of what they were eating (portion control) and how it affected their weight. For example, they reported eating healthier food and less food in total.

Adolescent and parent participants suggested that future interventions should include positive reinforcement with the self-weighing. For example, they suggested it would be beneficial to receive positive feedback when they are weighing at least a few times per week. They also indicated that they would welcome additional information from clinical staff, such as educational pointers sent via the weighing app about healthy habits.

##### 3) General reaction toward the self-weighing intervention:

###### Positive Reaction

Most adolescents (8 out of 11) and parents (6 out of 9) who participated in the interviews thought that the single-arm pilot study of having a clinic-connected scale at home was helpful. Most of the feedback from the participants was to increase the reminders to weigh in, add an exercise component that would encourage the adolescents to exercise, have colors to indicate a trend for when their child weighed themselves so the child would not be so caught up with the number, and make the setup of the scale easier.

While some adolescents found it stressful when parents asked about their daily weight status, they felt comfortable when their doctor asked about their weight. Additionally, other adolescents found it helpful or at least not stressful when their parents asked about their weight.

###### Neutral Reaction

The parents of the patients who felt neutral about the study reported their adolescent did not use the scale enough. The parents felt it was their responsibility to ensure that their child was using the scale every day, though indicated they did not tell their child to do so. For some children, this study encouraged them to exercise more often as they felt they were able to see the effect of exercise on their weight. For other children, this study had no effect on exercise as they were already involved in sports at school or they felt they were too busy to exercise.

During the interviews, the adolescents reported that weighing themselves regularly did affect their general outlook for the day – positive when their weight was down and negative when their weight was up. These participants reported the weighing as net neutral.

###### Negative Reaction

A small portion of the participants (2 out of 11) had negative reflections on the experience. These participants reported that they did not want to weigh when the desired results were not obtained. Parents felt that the adolescents worried about what the clinic thought of their weights and weighing frequency. They also felt that once-weekly weighing is frequent enough rather than the suggested once-daily.

## Discussion

This manuscript summarizes qualitative interview data from adolescents with obesity who prospectively enrolled into a self-weighing feasibility study using scales connected to the electronic health record. The objective of that feasibility study was to lay the groundwork for a randomized pilot using the EHR-connected scales. The objective of the qualitative interviews presented here was to describe the experiences that adolescents had with self-weighing on EHR-connected scales. If the qualitative interviews discovered overwhelming negative reactions from adolescents and parents to the self-weighing feasibility study, we would need to understand if harm outweighed risk before pursuing further study on this topic. Overall, the adolescents and their parents reported that the experience was positive enough to support the further study of remote self-monitoring of weight in adolescents with obesity who are seeking obesity treatment.

We were somewhat surprised that the interviewees suggested including more feedback and interaction between the clinic and the adolescents. The adolescent participants suggested that the clinic send recommendations to them based on the weights transmitted to the clinic from the weighing at home. Parents wanted specific suggestions about activity and dietary intake between clinic visits, and adolescents wanted more feedback on the weighing and weights. The most frequent suggestion from the parents and the adolescents was that there should be reminders to the adolescents to weigh at the recommended frequency.

In the context of Social Cognitive Theory (Fig. 1), it seemed that the adolescents and their parents highly valued the Personal Factors (feedback and expectations from clinic) that the Environmental Factors (scale provided by the single-arm feasibility study) made possible. While interviewees noted that the scales increased personal awareness, they noted a desire for specific guidance and knowledge for improving Behavioral factors. The adolescents also suggested that a future scale-based intervention should include prompts about exercising in the scale or self-weighing app. Other suggestions included color-coding to make it easier to see trends in weight. Connecting the scale was technologically difficult for many participants, and this should be addressed in future work.

One limitation of the study is the selection bias, which could limit generalizability. First, the feasibility sample was selected from adolescents seeking obesity treatment from a tertiary referral obesity clinic. Second, the feasibility study of self-weighing on blue-tooth scales connected to the EHR is a subset of the adolescents seeking obesity treatment at the clinic. Third, the interviews were conducted with adolescents who responded to a one-time invitation to complete a qualitative interview about the self-weighing experience. This is also a source of selection bias because adolescents who were willing to talk about their experiences were the ones who completed this 1:1 interview. These three sources of selection bias limit the generalizability of the findings beyond adolescents who are seeking obesity treatment. Future research could assess this form of self-monitoring in primary care-based samples.

## Conclusions

In summary, this study summarizes the experiences that adolescents seeking obesity treatment, and their parents had during a feasibility study of self-weighing on EHR-connected scales to monitor weight at home between clinic visits. These qualitative results affirm that this form of self-monitoring should be studied further in adolescents with obesity who are seeking obesity treatment. The population studied here may have unique perspectives that may not generalize to adolescents with obesity who are not seeking obesity treatment in a specialized pediatric obesity center.

## Supplementary Material

Supplementary Files

This is a list of supplementary files associated with this preprint. Click to download.

- Tables.docx

Tables

Tables 1 to 3 are available in the supplementary files section

## Data Availability

De-identified data will be stored according to data sharing policies and freely available in the Data Repository for U of M.

## Abbreviations

EHR: Electronic health record
IRB: Minnesota Institutional Review Board
BMI: Body mass index
